# Supplementary material for: Effects of company and season on blood fluke (Cardicola spp.) infection in ranched Southern Bluefin Tuna: preliminary evidence infection has a negative effect on fish growth
Source: PeerJ. 2023 Jul 25;11:e15763. doi: 10.7717/peerj.15763 (PMC10377432; doi:10.7717/peerj.15763)
Supplement: Supplemental Information 1 [file peerj-11-15763-s001.docx]

**Supplementary Table 1.** Prevalence (P) (95% confidence interval) of *Cardicola* spp. infection in ranched Southern Bluefin Tuna from Praziquantel treated pontoons sampled in July 2018, 2019 and 2021.

| **Company** | **Year** | **n** | **Adult *C. forsteri* in SBT heart** | ***C. forsteri* (ITS-2) in SBT heart** | ***Cardicola* spp. eggs in gills** | ***C. forsteri* (ITS-2) in SBT gills** | ***C. orientalis* (ITS-2) in SBT gills** |
| --- | --- | --- | --- | --- | --- | --- | --- |
|  |  |  | **P (%)** | **P (%)** | **P (%)** | **P (%)** | **P (%)** |
| A | 2018 | 14 | 93.3  (70.2-99.7) | 100  (79.6-100) | 100  (78.5-100) | 100  (78.5-100) | 7.14  (0.37-31.5) |
|  | 2019 | 15 | 66.7  (41.7-84.8) | 55.6  (26.7-81.1) | 86.7  (62.1-97.6) | 13.3  (2.37-37.9) | 0.00  (0.00-20.4) |
|  | 2021 | 12 | 0.00  (0.00-24.2) | 63.6  (35.4-84.8) | 58.3  (32.0-80.7) | 16.7  (2.96-44.8) | 0.00  (0.00-24.2) |
| B | 2018 | 15 | 26.7  (10.9-52.0) | 33.3  (15.2-58.3) | 80.0  (54.8-93.0) | 73.3  (48.0-89.1) | 6.67  (0.34-29.8) |
|  | 2019 | 15 | 28.6  (11.7-54.6) | 28.6  (11.7-54.6) | 73.3  (48.1-89.1) | 20.0  (7.05-45.2) | 0.00  (0.00-20.4) |
|  | 2021 | 15 | 53.3  (30.1-75.2) | 73.3  (48.1-89.1) | 66.7  (41.7-84.8) | 53.3  (30.1-75.2) | 0.00  (0.00-20.4) |
| C | 2018 | 15 | 42.9  (21.4-67.4) | 64.3  (38.8-83.7) | 86.7  (62.1-97.6) | 53.3  (30.1-75.2) | 0.00  (0.00-20.4) |
|  | 2019 | 15 | 14.3  (2.53-39.9) | 61.5  (35.5-82.3) | 73.3  (48.1-89.1) | 46.7  (24.8-69.9) | 0.00  (0.00-20.4) |
|  | 2021 | 15 | 13.3  (2.37-37.9) | 33.3  (15.2-58.3) | 53.3  (30.1-75.2) | 20.0  (7.05-45.2) | 0.00  (0.00-20.4) |
| D | 2018 | 15 | 53.3  (30.1-75.2) | 86.7  (62.1-97.6) | 100  (79.6-100) | 93.3  (70.2-99.7) | 0.00  (0.00-20.4) |
|  | 2019 | 15 | 20.0  (7.05-45.2) | 66.7  (41.7-84.8) | 73.3  (48.1-89.1) | 66.7  (41.7-84.8) | 6.67  (0.34-29.8) |
|  | 2021 | 14 | 6.67  (0.34-29.8) | 46.7  (24.8-69.9) | 71.4  (45.4-88.3) | 28.6  (11.7-54.6) | 0.00  (0.00-22.8) |
| E | 2018 | 15 | 50.0  (26.8-73.2) | 85.7  (60.1-97.5) | 100  (79.6-100) | 80.0  (54.8-92.3) | 0.00  (0.00-20.4) |
|  | 2019 | 15 | 20.0  (7.05-45.2) | 18.2  (3.23-47.7) | 46.7  (24.8-69.9) | 80.0  (54.8-92.3) | 0.00  (0.00-20.4) |
|  | 2021 | 15 | 69.2  (42.4-87.3) | 76.9  (49.7-91.8) | 93.3  (70.2-99.7) | 46.7  (24.8-69.9) | 0.00  (0.00-20.4) |
| F | 2018 | 15 | 46.7  (24.8-69.9) | 60.0  (35.7-80.2) | 80.0  (54.8-93.0) | 40.0  (19.8-64.3) | 0.00  (0.00-20.4) |
|  | 2019 | 14 | 42.9  (21.4-67.4) | 36.4  (15.2-64.6) | 78.6  (52.4-92.4) | 64.3  (38.8-83.7) | 0.00  (0.00-21.5) |
|  | 2021 | 15 | 66.7  (41.7-84.8) | 80.0  (54.8-93.0) | 80.0  (54.8-93.0) | 60.0  (35.7-80.2) | 0.00  (0.00-20.4) |
| G | 2018 | 13 | 40.0  (19.8-64.3) | 66.7  (41.7-84.8) | 84.6  (57.8-97.3) | 53.3  (30.1-75.2) | 0.00  (0.00-22.8) |
|  | 2019 | 15 | 73.3  (48.1-89.1) | 86.7  (62.1-97.6) | 100  (79.6-100) | 86.7  (62.1-97.6) | 13.3  (2.37-37.9) |
|  | 2021 | 15 | 26.7  (10.9-52.0) | 80.0  (54.8-93.0) | 73.3  (48.1-89.1) | 53.3  (30.1-75.2) | 0.00  (0.00-20.4) |
