# Supplementary material for: Effects of company and season on blood fluke (Cardicola spp.) infection in ranched Southern Bluefin Tuna: preliminary evidence infection has a negative effect on fish growth
Source: PeerJ. 2023 Jul 25;11:e15763. doi: 10.7717/peerj.15763 (PMC10377432; doi:10.7717/peerj.15763)
Supplement: Supplemental Information 2 [file peerj-11-15763-s002.docx]

**Supplementary Table 2.** Statistical differences at p ≤ 0.05 in prevalence of *Cardicola* spp. infection between companies for each year.

|  | 2018 | 2019 | 2021 |
| --- | --- | --- | --- |
| Adult *C. forsteri* | χ2 = 15.54, *p* = .0164 | χ2 = 21.26, *p* = .0016 | χ2 = 31.21, *p* < .0001 |
|  | A ↑ B (*p* = .0005)  A ↑ C (*p* = .0052)  A ↑ D (*p* = .0352)  A ↑ E (*p* = .0142)  A ↑ F (*p* = .0142)  A ↑ G (*p* = .0052) | A ↑ C (*p* = .0078)  A ↑ D (*p* = .0253)  A ↑ E (*p* = .0253)  G ↑ B (*p* = .0268)  G ↑ C (*p* = .0025)  G ↑ D (*p* = .0092)  G ↑ E (*p* = .0092) | B ↑ A (*p* = .0031)  B ↑ D (*p* = .0142)  E ↑ A (*p* = .0005)  E ↑ C (*p* = .0056)  E ↑ D (*p* = .0011)  F ↑ A (*p* = .0004)  F ↑ C (*p* = .0078)  F ↑ D (*p* = .0017) |
| *C. forsteri* (positive qPCR of ITS-2) in heart samples | χ2 = 20.99, *p* = .0018 | χ2 = 16.70, *p* = .0102 | χ2 = 16.51, *p* = .0113 |
|  | A ↑ B (*p* = .0002)  A ↑ C (*p* = .0169)  A ↑ F (*p* = .0169)  A ↑ G (*p* = .0421)  D ↑ B (*p* = .0078)  E ↑ B (*p* = .0078) | G ↑ B (*p* = .0063)  G ↑ E (*p* = .0012)  C ↑ E (*p* = .0472)  D ↑ E (*p* = .0214) | F ↑ C (*p* = .0253)  G ↑ C (*p* = .0078)  G ↑ D (*p* = .0209) |
| *Cardicola* spp. eggs in gill filaments |  | χ2 = 12.96, *p* = .0437 |  |
|  |  | G ↑ E (*p* = .0022) |  |
| *C. forsteri* (positive qPCR of ITS-2) in gill samples | χ2 = 21.16, *p* = .0017 | χ2 = 29.37, *p* < .0001 |  |
|  | A ↑ C (*p* = .0063)  A ↑ F (*p* = .0007)  A ↑ G (*p* = .0063)  D ↑ C (*p* = .0352)  D ↑ F (*p* = .0052)  D ↑ G (*p* = .0352) | D ↑ A (*p* = .0078)  D ↑ B (*p* = .0253)  E ↑ A (*p* = .0007)  E ↑ B (*p* = .0028)  F ↑ A (*p* = .0078)  F ↑ B (*p* = .0253)  G ↑ A (*p* = .0001)  G ↑ B (*p* = .0007) |  |
