# Supplementary material for: Effects of company and season on blood fluke (Cardicola spp.) infection in ranched Southern Bluefin Tuna: preliminary evidence infection has a negative effect on fish growth
Source: PeerJ. 2023 Jul 25;11:e15763. doi: 10.7717/peerj.15763 (PMC10377432; doi:10.7717/peerj.15763)
Supplement: Supplemental Information 3 [file peerj-11-15763-s003.docx]

**Supplementary Table 3.** Statistical differences at p ≤ 0.05 in prevalence of *Cardicola* spp. infection between years for each company. Note: no statistical differences seen for Company C or Company F.

|  | A | B | D | E | G |
| --- | --- | --- | --- | --- | --- |
| Adult *C. forsteri* | χ2 = 24.58, *p* < .0001 |  | χ2 = 8.864, *p* = .0119 | χ2 = 7.006,  *p* = .0301 | χ2 = 6.964,  *p* = .0307 |
|  | 2018 ↑ 2019 (*p* < .0001)  2018 ↑ 2021 (*p* < .0001) |  | 2018 ↑ 2021  (*p* = .0142) | 2021 ↑ 2019  (*p* = .0200) | 2019 ↑ 2021  (*p* = .0268) |
| *C. forsteri* (positive qPCR of ITS-2) in heart samples | χ2 = 7.691,  *p* = .0187 | χ2 = 7.201,  *p* = .0273 | χ2 = 7.177,  *p* = .0276 | χ2 = 13.68,  *p* = .0011 |  |
|  | 2018 ↑ 2019 (*p* = .0119) 2018 ↑ 2021 (*p* = .0221) | 2021 ↑ 2019 (*p* = .0268) | 2018 ↑ 2021  (*p* = .0209) | 2018 ↑ 2019  (*p* = .0012)  2021 ↑ 2019 (*p* = .0123) |  |
| *Cardicola* spp. eggs in gill filaments | χ2 = 8.157,  *p* = .0169 |  |  | χ2 = 15.83,  *p* = .0004 |  |
|  | 2018 ↑ 2021 (*p* = .0120) |  |  | 2018 ↑ 2019  (*p* = .0022)  2021 ↑ 2019 (*p* = .0142) |  |
| *C. forsteri* (positive qPCR of ITS-2) in gill samples | χ2 = 27.19,  *p* < .0001 | χ2 = 8.715,  *p* = .0128 | χ2 = 13.21,  *p* = .0014 |  |  |
|  | 2018 ↑ 2019  (*p* < .0001)  2018 ↑ 2021  (*p* < .0001) | 2018 ↑ 2019  (*p* = .0014) | 2018 ↑ 2021  (*p* = .0005) |  |  |
