# Supplementary material for: Effects of company and season on blood fluke (Cardicola spp.) infection in ranched Southern Bluefin Tuna: preliminary evidence infection has a negative effect on fish growth
Source: PeerJ. 2023 Jul 25;11:e15763. doi: 10.7717/peerj.15763 (PMC10377432; doi:10.7717/peerj.15763)
Supplement: Supplemental Information 4 [file peerj-11-15763-s004.docx]

**Supplementary Table 2.** Mean intensity (I) (±SE) of *Cardicola* spp. infection in ranched Southern Bluefin Tuna from Praziquantel treated pontoons sampled in July 2018, 2019 and 2021. n.a – not applicable.

| **Company** | **Year** | **n** | **Adult *C. forsteri* in SBT heart** | ***C. forsteri* (ITS-2) in SBT heart** | ***Cardicola* spp. eggs in gills** | ***C. forsteri* (ITS-2) in SBT gills** | ***C. orientalis* (ITS-2) in SBT gills** |
| --- | --- | --- | --- | --- | --- | --- | --- |
|  |  |  | **I** | **I** | **I** | **I** | **I** |
| A | 2018 | 14 | 4.00  (±0.80) | 5.29 x 10^6^  (±1.17 x 10^6^) | 1.33  (±0.23) | 1.07 x 10^7^ (±3.97 x 10^6^) | 3.23 x 10^6^  (n.a) |
|  | 2019 | 15 | 2.71  (±0.68) | 2.91 x 10^5^  (±1.39 x 10^5^) | 0.13  (±0.04) | 2.61 x 10^6^ (±5.26 x 10^5^) | - |
|  | 2021 | 12 | 0.00  (±0.00) | 1.23 x 10^6^  (±3.98 x 10^5^) | 0.33  (±0.11) | 5.68 x 10^5^ (±3.03 x 10^5^) | - |
| B | 2018 | 15 | 1.75  (±0.75) | 1.58 x 10^5^  (±3.44 x 10^4^) | 0.43  (±0.20) | 1.17 x 10^6^ (±2.80 x 10^5^) | 2.33 x 10^6^  (n.a) |
|  | 2019 | 15 | 1.50  (±0.29) | 5.07 x 10^4^)  (±1.98 x 10^4^) | 0.17  (±0.06) | 9.41 x 10^4^ (±4.60 x 10^4^) | - |
|  | 2021 | 15 | 1.38  (±0.18) | 7.97 x 10^5^)  (±3.78 x 10^5^) | 0.54  (±0.17) | 2.64 x 10^6^ (±6.18 x 10^5^) | - |
| C | 2018 | 15 | 1.17  (±0.17) | 4.58 x 10^4^)  (±6.87 x 10^3^) | 0.22  (±0.08) | 5.23 x 10^5^  (±1.74 x 10^5^) | - |
|  | 2019 | 15 | 1.00  (±0.00) | 8.56 x 10^5^)  (±4.46 x 10^5^) | 0.60  (±0.22) | 4.19 x 10^5^ (±2.03 x 10^5^) | - |
|  | 2021 | 15 | 1.00  (±0.00) | 2.23 x 10^5^  (±1.92 x 10^5^) | 0.45  (±0.28) | 3.81 x 10^6^ (±1.45 x 10^6^) | - |
| D | 2018 | 15 | 1.63  (±0.38) | 4.96 x 10^5^  (±2.39 x 10^5^) | 1.00  (±0.23) | 2.97 x 10^6^  (±5.45 x 10^5^) | - |
|  | 2019 | 15 | 1.00  (±0.00) | 1.17 x 10^6^  (±5.11 x 10^5^) | 0.45  (±0.21) | 3.15 v 10^5^ (±2.37 x 10^5^) | 1.07 x 10^7^  (n.a) |
|  | 2021 | 14 | 2.00  (±0.00) | 2.13 x 10^5^  (±1.05 x 10^5^) | 0.27  (±0.07) | 6.71 x 10^6^ (±2.82 x 10^6^) | - |
| E | 2018 | 15 | 3.00  (±0.76) | 9.58 x 10^4^  (±6.87 x10^4^) | 0.66  (±25) | 1.39 x 10^6^  (±4.72 x 10^5^) | - |
|  | 2019 | 15 | 1.67  (±0.33) | 1.11 x 10^5^  (±6.38 x 10^3^) | 0.11  (±0.02) | 1.22 x 10^5^ (±2.66 x 10^4^) | - |
|  | 2021 | 15 | 2.67  (±1.21) | 3.92 x 10^5^  (±1.86 x 10^5^) | 0.28  (±0.07) | 3.19 x 10^6^  (±9.14 x 10^5^) | - |
| F | 2018 | 15 | 1.29  (±0.18) | 6.59 x 10^4^  (±2.60 x 10^4^) | 0.29  (±0.14) | 3.47 x 10^5^  (±8.12 x 10^4^) | - |
|  | 2019 | 14 | 1.50  (±0.22) | 2.43 x 10^5^  (±1.05 x 10^4^) | 0.18  (±0.05) | 9.02 x 10^5^ (±2.92 x 10^5^) | - |
|  | 2021 | 15 | 1.90  (±0.35) | 4.12 x 10^5^  (±1.10 x 10^5^) | 0.24  (±0.05) | 3.78 x 10^6^ (±1.26 x 10^6^) | - |
| G | 2018 | 13 | 1.50  (±0.34) | 2.59 x 10^5^  (±6.62 x 10^4^) | 0.13  (±0.04) | 6.36 x 10^5^  (±2.36 x 10^5^) | - |
|  | 2019 | 15 | 4.73  (±1.00) | 3.19 x 10^6^  (±1.56 x 10^6^) | 1.03  (±0.37) | 3.85 x 10^6^ (±9.29 x 10^5^) | 1.05 x 10^5^  (±1.50 x 10^4^) |
|  | 2021 | 15 | 1.25  (±0.25) | 3.41 x 10^5^  (±1.44 x 10^5^) | 0.33  (±0.05) | 4.71 x 10^6^ (±2.02 x 10^6^) | - |
