# Supplementary material for: Effects of company and season on blood fluke (Cardicola spp.) infection in ranched Southern Bluefin Tuna: preliminary evidence infection has a negative effect on fish growth
Source: PeerJ. 2023 Jul 25;11:e15763. doi: 10.7717/peerj.15763 (PMC10377432; doi:10.7717/peerj.15763)
Supplement: Supplemental Information 5 [file peerj-11-15763-s005.docx]

**Supplementary Table 5.** Statistical differences at p ≤ 0.05 in mean intensity of *Cardicola* spp. infection between companies for each year.

|  | 2018 | 2019 | 2021 |
| --- | --- | --- | --- |
| Adult *C. forsteri* |  |  | H = 27.68, p = .0001 |
|  |  |  | A ↑ B (*p* = .0050)  A ↑ E (*p* = .0013)  A ↑ F (*p* = .0001)  A ↑ G (*p* = .0490) |
| *C. forsteri* (ITS-2) in heart samples | H = 46.22, *p* < .0001 | H = 12.74, *p* = .0473 |  |
|  | A ↑ B (*p* = .0002)  A ↑ C (*p* < .0001)  A ↑ D (*p* = .0153)  A ↑ E (*p* < .0001)  A ↑ F (*p* < .0001) | G ↑ B (*p* = .0220) |  |
| *Cardicola* spp. eggs in gill filaments | H = 33.40, *p* < .0001 | H = 18.47, *p* = .0052 |  |
|  | A ↑ C (*p* = .0018)  A ↑ F (*p* = .0042)  A ↑ G (*p* = .0005)  D ↑ C (*p* = .0354)  D ↑ G (*p* = .0107) | G ↑ A (*p* = .0094) |  |
| *C. forsteri* (ITS-2) in gill samples | H = 39.50, *p* < .0001 | H = 35.71, *p* < .0001 |  |
|  | A ↑ B (*p* = .0103)  A ↑ C (*p* = .0002)  A ↑ E (*p* = .0051)  A ↑ F (*p* = .0004)  A ↑ G (*p* = .0003)  D ↑ C (*p* = .0499) | G ↑ B (*p* = .0319)  G ↑ D (*p* < .0001)  G ↑ E (*p* < .0001) |  |
