# Supplementary material for: Effects of company and season on blood fluke (Cardicola spp.) infection in ranched Southern Bluefin Tuna: preliminary evidence infection has a negative effect on fish growth
Source: PeerJ. 2023 Jul 25;11:e15763. doi: 10.7717/peerj.15763 (PMC10377432; doi:10.7717/peerj.15763)
Supplement: Supplemental Information 6 [file peerj-11-15763-s006.docx]

**Supplementary Table 6.** Statistical differences at p ≤ 0.05 in mean intensity of *Cardicola* spp. infection between years for each company.

|  | A | B | C | D | E | F | G |
| --- | --- | --- | --- | --- | --- | --- | --- |
| Adult  *C. forsteri* | H = 20.86,  *p* < .0001 |  |  |  |  |  | H = 8.341,  *p* = .0040 |
|  | 2018 ↑ 2021 (*p* < .0001)  2019 ↑ 2021 (*p* = .0040) |  |  |  |  |  | 2019 ↑ 2018  (*p* = .0225)  2019 ↑ 2021  (*p* = .0141) |
| *C. forsteri* (ITS-2) in heart samples | H = 15.11,  *p* = .0005 | H = 6.301,  *p* = .0348 | H = 7.831,  *p* = .0140 |  | H = 9.732,  *p* = .0077 |  | H = 8.456,  *p* = .0146 |
|  | 2018 ↑ 2019 (*p* = .0009) 2018 ↑ 2021 (*p* = .0494) | 2021 ↑ 2019 (*p* = .0365) | 2019 ↑ 2018  (*p* = .0316) |  | 2021 ↑ 2018  (*p* = .0080) | 2021 ↑ 2018  (*p* = .0067) | 2019 ↑ 2021  (*p* = .0140) |
| *Cardicola* spp. eggs in gill filaments | H = 7.831,  *p* = .0140 |  |  |  | H = 7.087,  *p* = .0289 |  | H = 12.19,  *p* = .0023 |
|  | 2018 ↑ 2019  (*p* < .0001)  2018 ↑ 2021  (*p* = .0134) |  |  |  | 2018 ↑ 2019  (*p* = .0302) |  | 2019 ↑ 2018  (*p* = .0021) 2019 ↑ 2021 (*p* = .0350) |
| *C. forsteri* (ITS-2) in gill samples |  | H = 9.220,  *p* = .0039 | H = 6.229,  *p* = .0341 | H = 16.71,  *p* = .0002 | H = 19.31,  *p* < .0001 | H = 7.738,  *p* = .0209 | H = 10.51,  *p* = .0052 |
|  |  | 2021 ↑ 2019  (*p* = .0093) | 2021 ↑ 2019  (*p* = .0422) | 2018 ↑ 2019  (*p* = .0014)  2021 ↑ 2019  (*p* = . 0024) | 2018 ↑ 2019  (*p* = .0043)  2021 ↑ 2019  (*p* = .0001) | 2021 ↑ 2018  (*p* = .0261) | 2019 ↑ 2018  (*p* = .0054)  2021 ↑ 2018  (*p* = .0410) |
